# Midgut volvulus due to congenital malrotation in an adult: a case report

**DOI:** 10.1186/s13256-023-04096-5

**Published:** 2023-08-25

**Authors:** Almuntasir beallah Eltayb, Albra Hegazi, Osaman Elhag, Amro Abdelgadir

**Affiliations:** 1 Midland Regional Hospital Mullingar, Mullingar, Ireland; 2 Sudan Medical Specialization broad, Al Khurtum, Sudan

**Keywords:** Midgut Malrotation, Volvulus, Intestinal obstruction, Ladd procedure

## Abstract

**Background:**

Intestinal Malrotation is congenital that complicates 1 in every 200 births. It results from abnormal fixation and rotation of the gut tube during fetal development. It is usually asymptomatic and most cases are diagnosed during childhood and the condition is seldom seen in adults. Also, it can present as an incidental finding in imaging or patients undergoing laparotomy for other reasons. Midgut Volvulus can lead to small obstruction it warrants surgical intervention.

**Case presentation:**

Our case is a 20 years old black African male from Khartoum (Sudan) who presented to the emergency department with features of intestinal obstruction he was resuscitated and underwent a CT scan of the abdomen that showed malrotation of the intestine with volvulus for which he underwent a Ladd procedure.

**Conclusion:**

Small bowel obstruction due to volvulus complicating malrotation is a rare presentation in adulthood and the Ladd procedure can be utilized to manage such case.

## Introduction

The intestine is made of foregut, midgut and hindgut. During the fourth week of embryonic development the midgut increases in length and the abdominal cavity can’t accommodate it and so it herniates through the umbilicus. During the tenth week of development, the midgut returns back to the abdomen and while doing so it rotates a 270° counter clockwise around the axis of the superior mesenteric artery, which supplies it so that the right colon is placed on the right side of the body. Failure of this process results in the formation of the midgut malrotation. The exact cause is not yet established, but there is often a fibrous band(ladd band) that tethers the right colon, preventing it from rotation and can lead to symptoms of obstruction [1].

## Case presentation

**Fig. 1:**
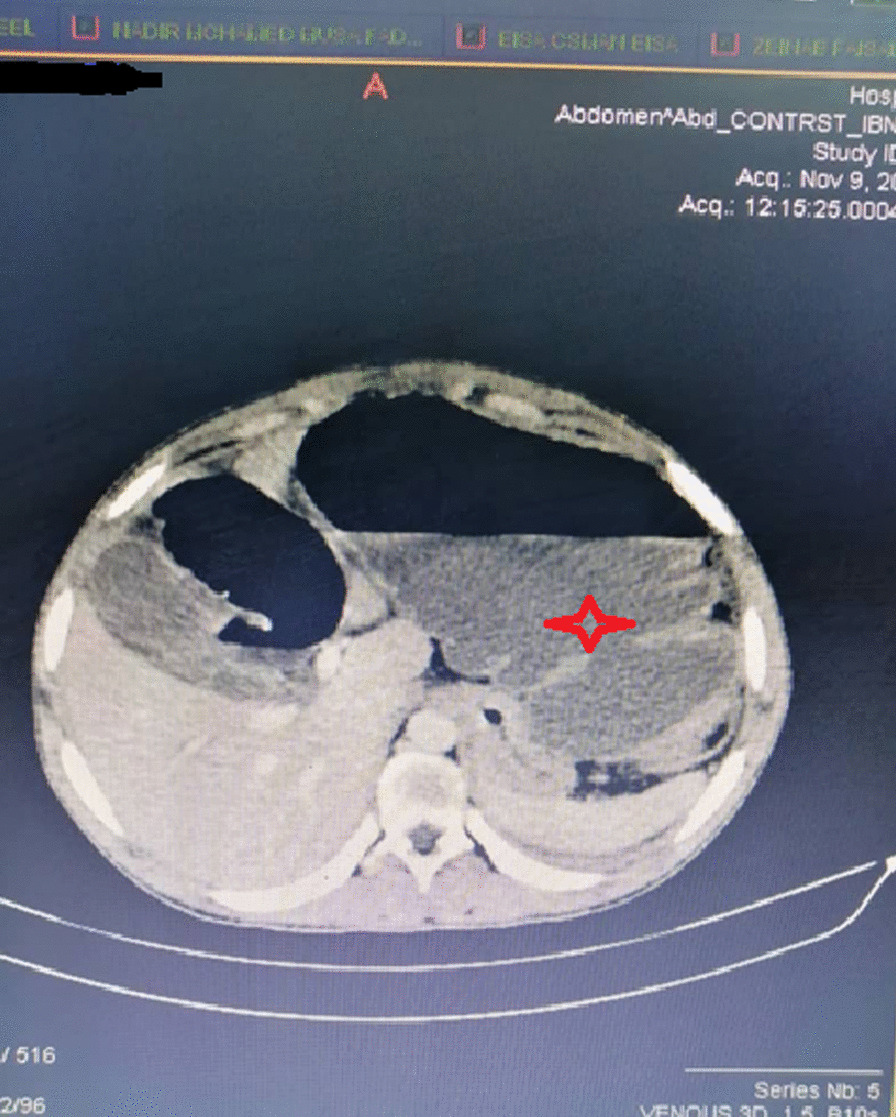
Dilated cecum in left-sided (red star)

**Fig. 2:**
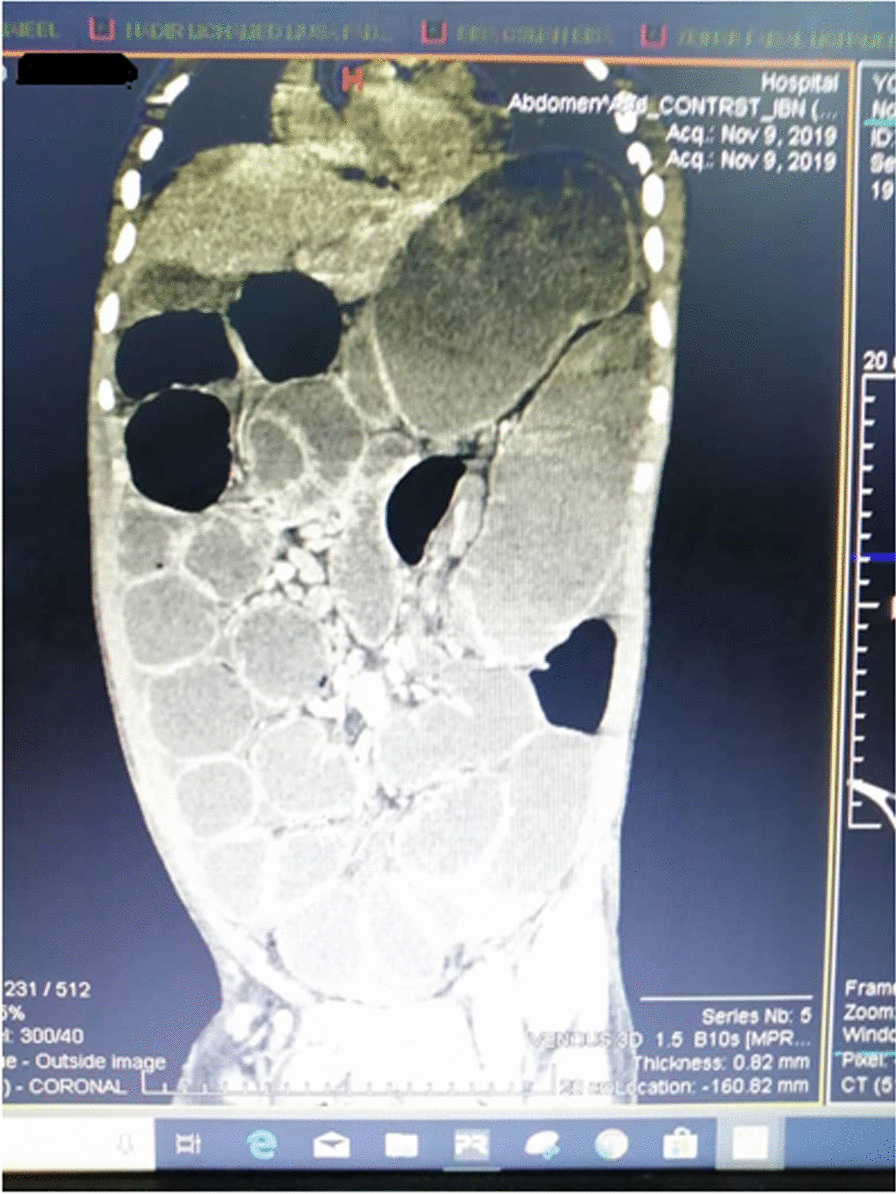
Dilated cecum in left-sided

A 20-year-old black African male from Sudan presented to Omdurman teaching hospital Emergency department complaining of acute onset colicky abdominal pain the patient passed flatus and feces 48 h prior to his presentation. also had vomiting and abdominal distension. He had a clear medical background with no history of operation before. After the initial assessment positive findings were abdominal distension, hyper-resonance in percussion, and exacerbated bowel sound, all blood lab results were normal. Management was started at the emergency department with a nasogastric tube and urinary catheter insertion. Intravenous fluids, broad-spectrum antibiotics. Abdominal X-ray showed multiple air-fluid levels and dilated loops of the bowel. Chest X-ray did not show air under the diaphragm.An emergency computed tomography (CT) scan was obtained which demonstrated dilated caecum was located in the left upper quadrant and small-bowel volvulus (Figs. 1, 2). There were also a few dilated loops of small bowel in the upper abdomen. The decision was made to emergency laparotomy.

**Fig. 3:**
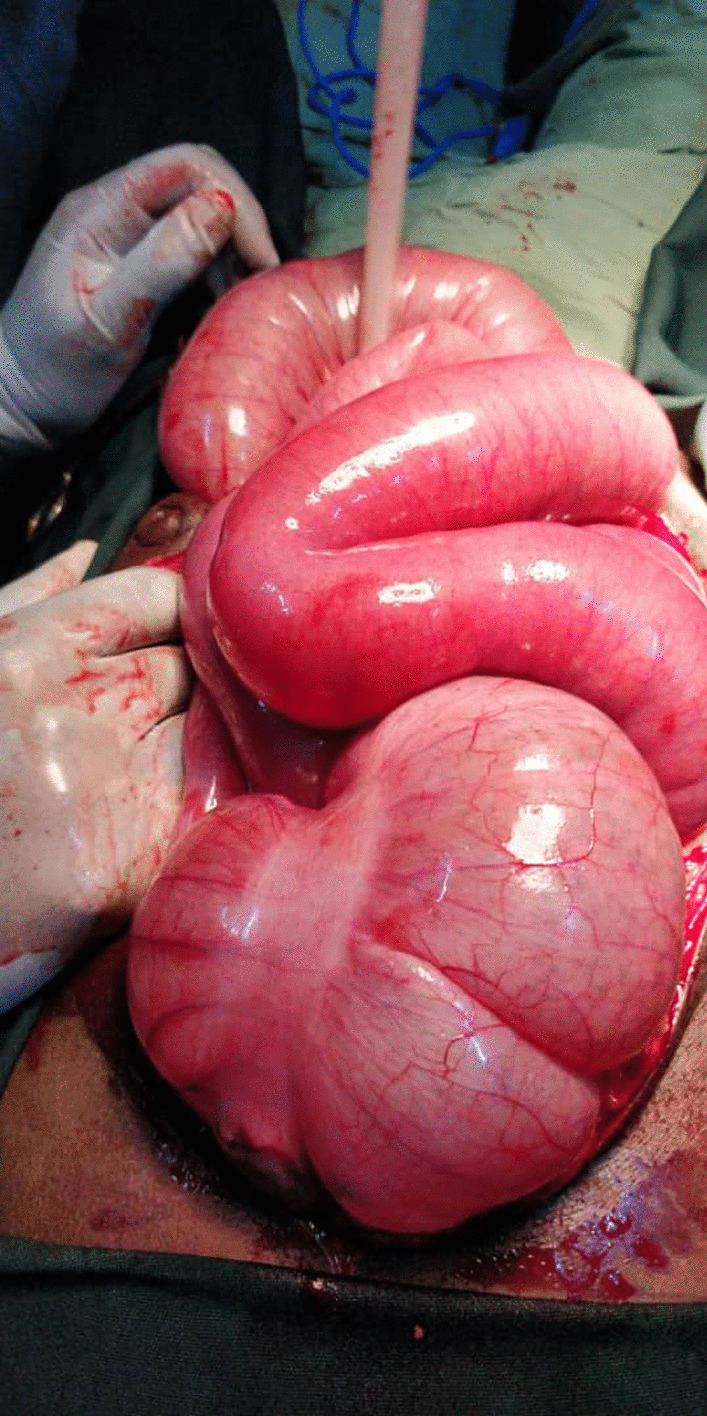
Dilated cecum and small-bowel

**Fig. 4:**
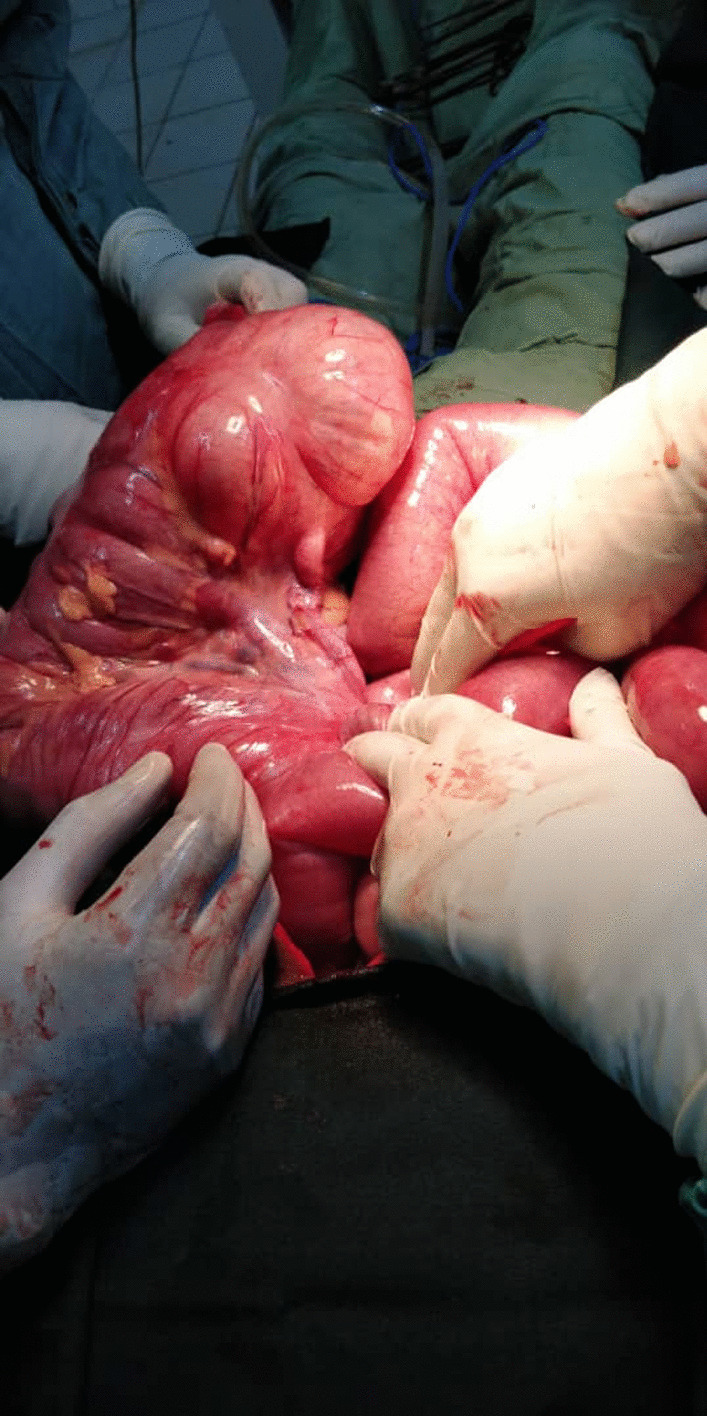
Cecum in the left

The findings of the operation included dilated small bowel in the upper abdomen and a dilated caecum found on the left side of the abdomen, DJ flexure on the right side (Figs. 3, 4). There was also necrosis of 155 cm of the small bowel just 56 cm from DJ flexure with one degree of twisting. Loops of small bowel occupying the right paracolic gutter and the right iliac fossa. There were fibrous bands over the distal part of the duodenum, on the right side of the abdomen, confirming midgut malrotation. The twisted necrotic small bowel was resected and anastomosis was performed, the congenital band was divided, the mesentery was broadened and an appendectomy was carried out with correction of the anatomical malrotation. The operation was carried out by a consultant and senior registrars who were exposed to malrotation surgery during the pediatrics surgery shift.

The patient had an uneventful postoperative recovery. He was followed up one month later in the outpatient department and he had no complaints and returned to his normal baseline.

## Discussion

Abnormal bowel rotation during Embryogenesis leads to the development of Malrotation and there different classification for the condition based on the degree of the rotation. According to stinger classification, there are three types of malrotation. Type 1: non-rotation, type 2: duodenal malrotation and type 3: duodenal plus caecal malrotation. Also, there is malfixation of the mesentery with a narrow pedicle that can result in volvulus. Several conditions are associated with midgut malrotation like Martinez-Friay syndrome where malrotation is associated with multiple GI atresia [2].

In pediatric patients, intestinal malrotation presents early with symptoms of small bowel obstruction ((abdominal distension and bilious vomiting) and can be diagnosed with upper GI contrast series. However, most adults with malrotation are often diagnosed accidentally during imaging or exploratory laparotomy for other causes [3]. Many adult patients might be asymptomatic or have nonspecific GI symptoms like abdominal pain only. Malrotation in adults is a rare cause of intestinal obstruction and its rate in autopsy is 1 in 6000 [4].

Both CT scans and upper GI series can help to detect malrotation. They can show the abnormal position of the caecum on the left and the abnormal relationship of the Superior mesenteric artery and superior mesenteric vein and SMV (the artery is found to the left of the vein in malrotation). Midgut volvulus is easier to be found in the upper GI series (shows corkscrew sign of the proximal small bowel), but because most adults undergo a contrast CT scan you might find a whirlpool appearance (swirling appearance of bowel and mesentery twisted around the axis of the superior mesenteric artery). Other features are duodenal obstruction and mesenteric vasculature congestion [5].

Children and adults who were found to have symptomatic malrotation should undergo surgery [6]. ladd's operation is the treatment of choice in both children and adults [7]. Ladd’s operation involves counter-clockwise detorsion of the small bowel, surgical division of ladd's band(fibrous tissue that tethers the intestine) widening of small bowel mesentery, performing appendectomy and replacement of the small bowel to the right and caecum and colon to the left. It can done via midline incision or laparoscopy. Despite limitations, some authors had concluded that the use of laparoscopy in treating cases of malrotation is safe and effective [8]. However, in cases presenting with volvulus managing it with laparoscopy can be impossible and it’s safe to operate via a midline incision [7].

## Conclusion

Malrotation presenting with small bowel obstruction in adults is a rare presentation of the condition can be difficult to diagnosed clinically. Imaging is of great use to allow diagnoses and pre-operative planning. Ladd procedure can be carried out in the in adult as well but sometimes necrotic may need to be resected to complete the procedure.

## Data Availability

Availability of data is present in the reference section and its openly available.
